# Supplementary material for: Evidence supported by Mendelian randomization: impact on inflammatory factors in knee osteoarthritis
Source: Front Med (Lausanne). 2024 May 28;11:1382836. doi: 10.3389/fmed.2024.1382836 (PMC11165061; doi:10.3389/fmed.2024.1382836)
Supplement: Supplementary file 1 [file Data_Sheet_1.ZIP › data/GCST90274802_5e6/802.leaveoneout.pdf]

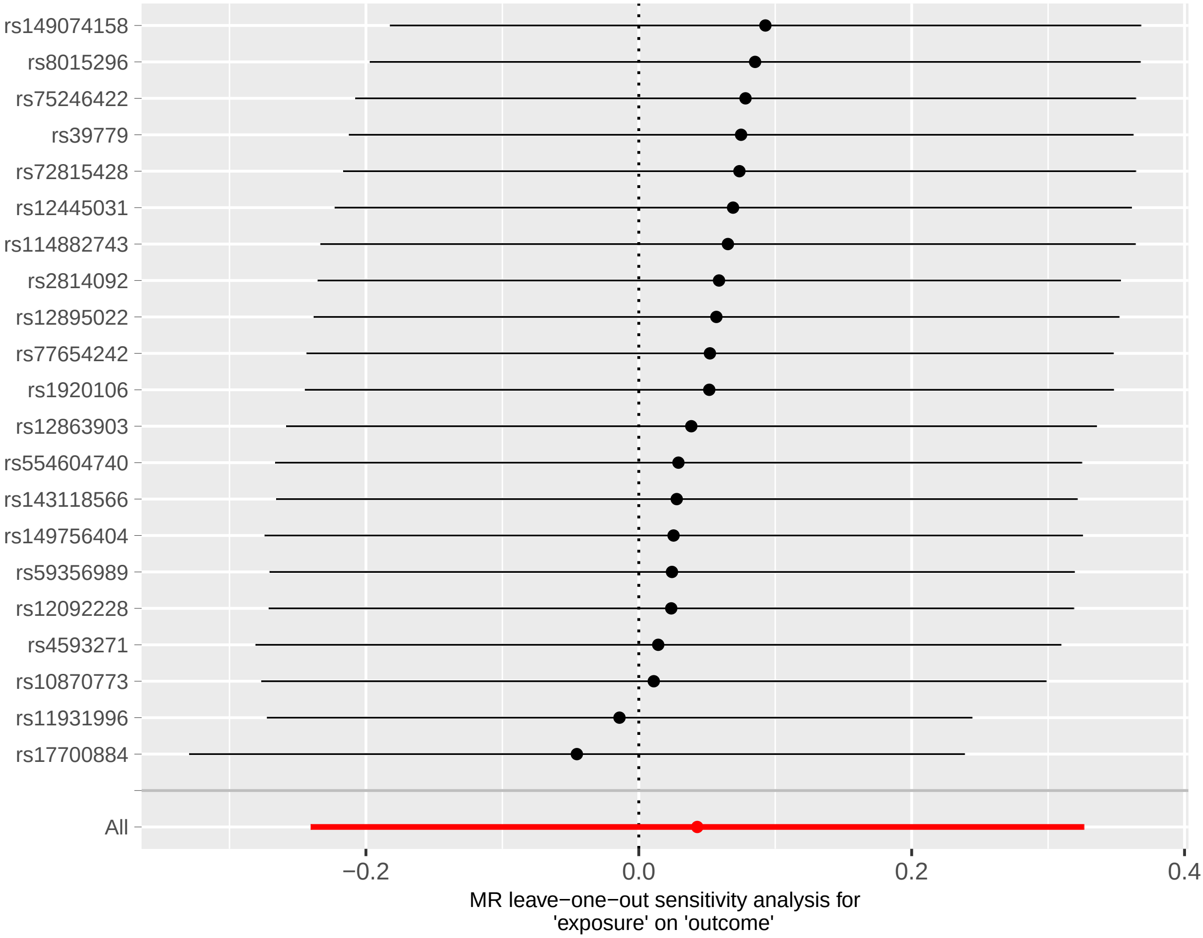
rs149074158
rs8015296
rs75246422
rs39779
rs72815428
rs12445031
rs114882743
rs2814092
rs12895022
rs77654242
rs1920106
rs12863903
rs554604740
rs143118566
rs149756404
rs59356989
rs12092228
rs4593271
rs10870773
rs11931996
rs17700884
All
−0.2
0.0
0.2
0.4
MR leave−one−out sensitivity analysis for
'exposure' on 'outcome'
